# Targeting the chromatin remodelling protein Brahma‐related gene 1 for intervention of pulmonary fibrosis

**DOI:** 10.1002/ctm2.1775

**Published:** 2024-08-21

**Authors:** Teng Wu, Bingshu Wang, Xianhua Gui, Ruiqi Liu, Dong Wei, Yong Xu, Shaojiang Zheng, Nan Li, Ming Kong

**Affiliations:** ^1^ Departments of Pathophysiology and Human Anatomy Key Laboratory of Targeted Intervention of Cardiovascular Disease and Collaborative Innovation Center for Cardiovascular Translational Medicine Nanjing Medical University Nanjing China; ^2^ Key Laboratory of Emergency and Trauma of Ministry of Education Engineering Research Center for Hainan Biological Sample Resources of Major Diseases Hainan Clinical Medical Center, the First Affiliated Hospital of Hainan Medical University Haikou China; ^3^ Department of Pathology the Second Affiliated Hospital of Hainan Medical University Haikou China; ^4^ Department of Respiratory Medicine Affiliated Nanjing Drum Tower Hospital Nanjing University School of Medicine Nanjing China; ^5^ Department of Lung Transplantation Wuxi People's Hospital Affiliated with Nanjing Medical University Wuxi China; ^6^ Department of Pharmacology State Key Laboratory of Natural Medicines China Pharmaceutical University Nanjing China

Dear Editor,

We describe in this letter a novel mechanism whereby the chromatin remodelling protein Brahma‐related gene 1 (BRG1) contributes to pulmonary fibrosis.

Pulmonary fibrosis is a common manifestation of interstitial lung disease (ILD) that affects over 40 million people worldwide.^1^ Although for a majority of patients with pulmonary fibrosis, one or another underlying cause including radiation, hypersensitivity pneumonitis, and pneumoconiosis have been identified, pulmonary fibrosis can occur in certain individuals with no ascribable aetiology; the latter patient group is categorized as idiopathic pulmonary fibrosis (IPF).^2^ Regardless of aetiology, extracellular matrix (ECM)‐producing myofibroblasts is the principal mediator of pulmonary fibrosis.^3^ Compared to quiescent fibroblasts from which they are derived, myofibroblasts are highly proliferative and migratory, able to perform muscle‐like contraction, and markedly more potent in producing ECM proteins.^4^ BRG1 is part of the epigenetic machinery that shapes the transcriptomic landscape in mammalian cells.^5^ In the present study, we sought to determine the role of BRG1 in pulmonary fibrosis.

In the first set of experiments, C57/BL6 mice were given bleomycin to induce pulmonary fibrosis followed by isolation of primary pulmonary fibroblasts. Rapid induction of both BRG1 and periostin, a marker for mature myofibroblast, was observed in the fibroblasts isolated from the lungs 1 week after bleomycin instillation (Figure S1A,B). When primary murine pulmonary fibroblasts or human pulmonary fibroblasts (MRC5) were exposed to transforming growth factor‐β (TGF‐β), BRG1 expression was up‐regulated with a similar kinetics as periostin (Figure S1C,D). BRG1 levels were substantially elevated in the lung tissues of IPF patients compared to the healthy individuals (Figure S1E). In addition, a significant correlation was identified between BRG1 expression and periostin expression (Figure S1F).

Next, primary pulmonary fibroblasts were isolated from BRG1^f/f^ mice and induced to differentiate into myofibroblasts by TGF‐β treatment; BRG1 deletion by transduction with Cre‐delivering adenovirus significantly attenuated myofibroblast marker genes (Figure 1A), cell proliferation (Figure 1B), cell migration (Figure 1C), and cell contraction (Figure 1D). Similarly, BRG1 knockdown by small interfering RNAs in pulmonary fibroblasts from IPF patients markedly decreased myofibroblast marker gene expression and attenuated cell proliferation/migration/contraction (Figure S2). To verify whether BRG1 deletion in myofibroblasts would alter pulmonary fibrosis in vivo, BRG1^f/f^ mice were crossbred with *Postn*‐Cre^ERT2^ mice to generate myofibroblast conditional BRG1 knockout mice (BRG1^ΔMF^, Figure 1E). Pulmonary fibrosis, as measured by Picrosirius Red staining and Masson's staining, was significantly dampened by BRG1 deletion in myofibroblasts (Figure 1F). In addition, measurements of myofibroblast marker genes (Figure 1G) and hydroxyproline quantification (Figure 1H) confirmed that the BRG1^ΔMF^ mice developed less severe pulmonary fibrosis than the BRG1^f/f^ mice. Notably, pulmonary inflammation was comparable between the BRG1^ΔMF^ mice and the BRG1^f/f^ mice (Figure S3).

**FIGURE 1:**
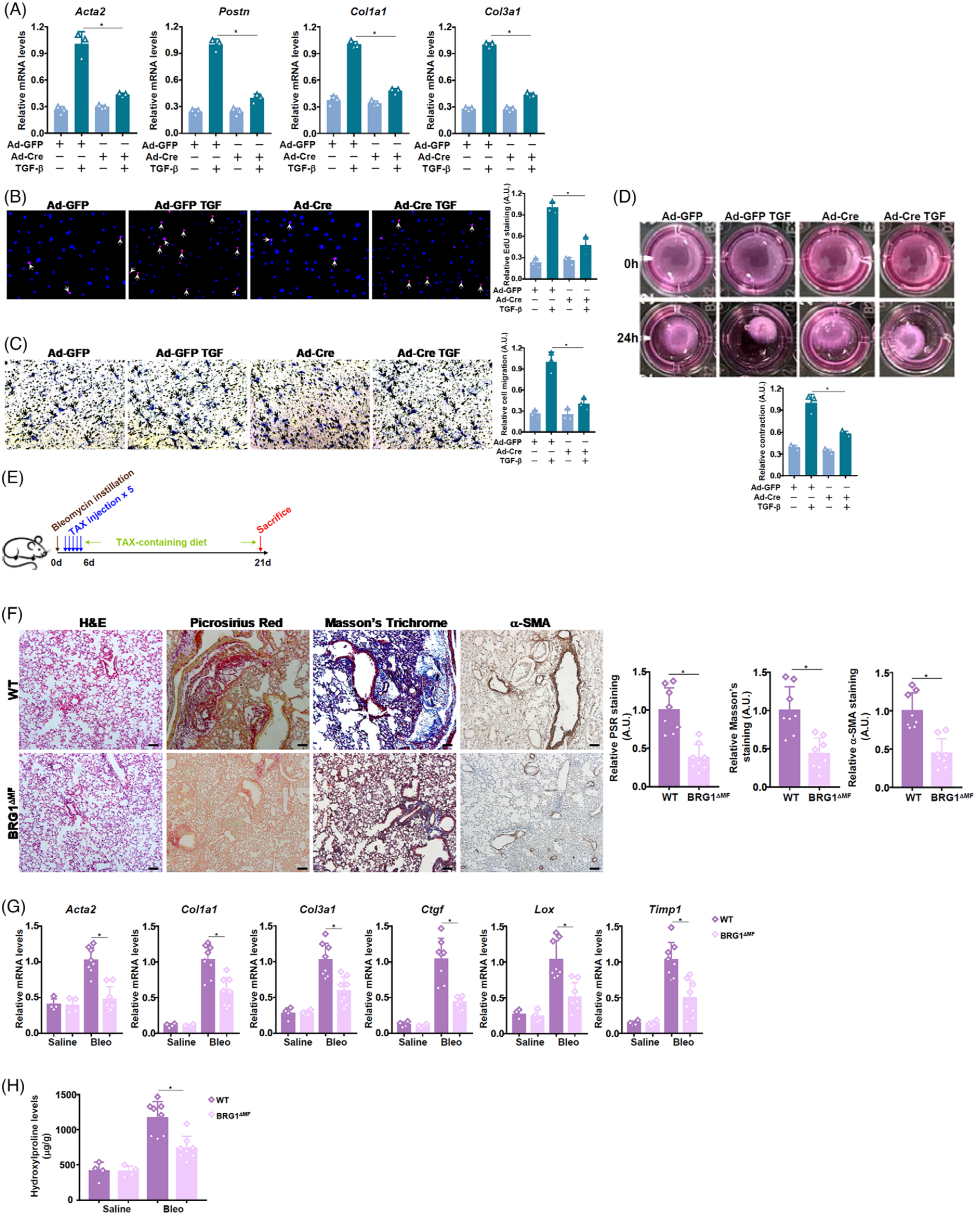
Brahma‐related gene 1 (BRG1) ablation attenuates fibroblast‐myofibroblast transition in vitro and pulmonary fibrosis in mice. (A–D) Pulmonary fibroblasts were isolated from BRG1^f/f^ mice and transduced with indicated adenovirus followed by treatment with transforming growth factor‐β (TGF‐β) (5 ng/mL) for 24 h. Myofibroblast marker genes were examined by quantitative polymerase chain reaction (qPCR) (A). EdU incorporation (B). Boyden transwell (C). Collagen contraction assay (D). *N* = 3 biological replicates. Data are expressed as mean ± S.D. *, *p*<0.05, one‐way analysis of variance (ANOVA) with post‐hoc Scheff´e. (E–H) Myofibroblast conditional BRG1 deletion mice (BRG1^ΔMF^) and wild‐type (WT) control mice were subjected to bleomycin instillation to induce pulmonary fibrosis. Scheme of experimental protocol (E). Paraffin sections were stained with Picrosirius Red and Masson's trichrome (F). Pro‐fibrogenic genes were examined by qPCR (G). Hydroxyproline levels (H). *N* = 5 ice for each group. Data are expressed as mean ± S.D. *, *p*<0.05, one‐way ANOVA with post‐hoc Scheff´e.

In primary murine pulmonary fibroblasts, the addition of PFI‐3, a small‐molecule BRG1 inhibitor that targets the bromodomain (BRD) of BRG1^6^ dose‐dependently attenuated fibroblast‐myofibroblast transition (Figure 2A‐‐D). It was also observed that PFI‐3 treatment led to a marked decrease in myofibroblast marker gene expression and cell proliferation/migration/contraction (Figure S4) in IPF cells. PFI‐3 administration, as an interventional approach (Figure 2E), suppressed pulmonary fibrosis in mice (Figure 2F‐‐H). It is noteworthy that PFI‐3 administration led to a significant decrease in immune cell infiltration and expression levels of interleukin (IL)‐6 and iNOS but not IL‐1β or TNF‐α (Figure S5).

**FIGURE 2:**
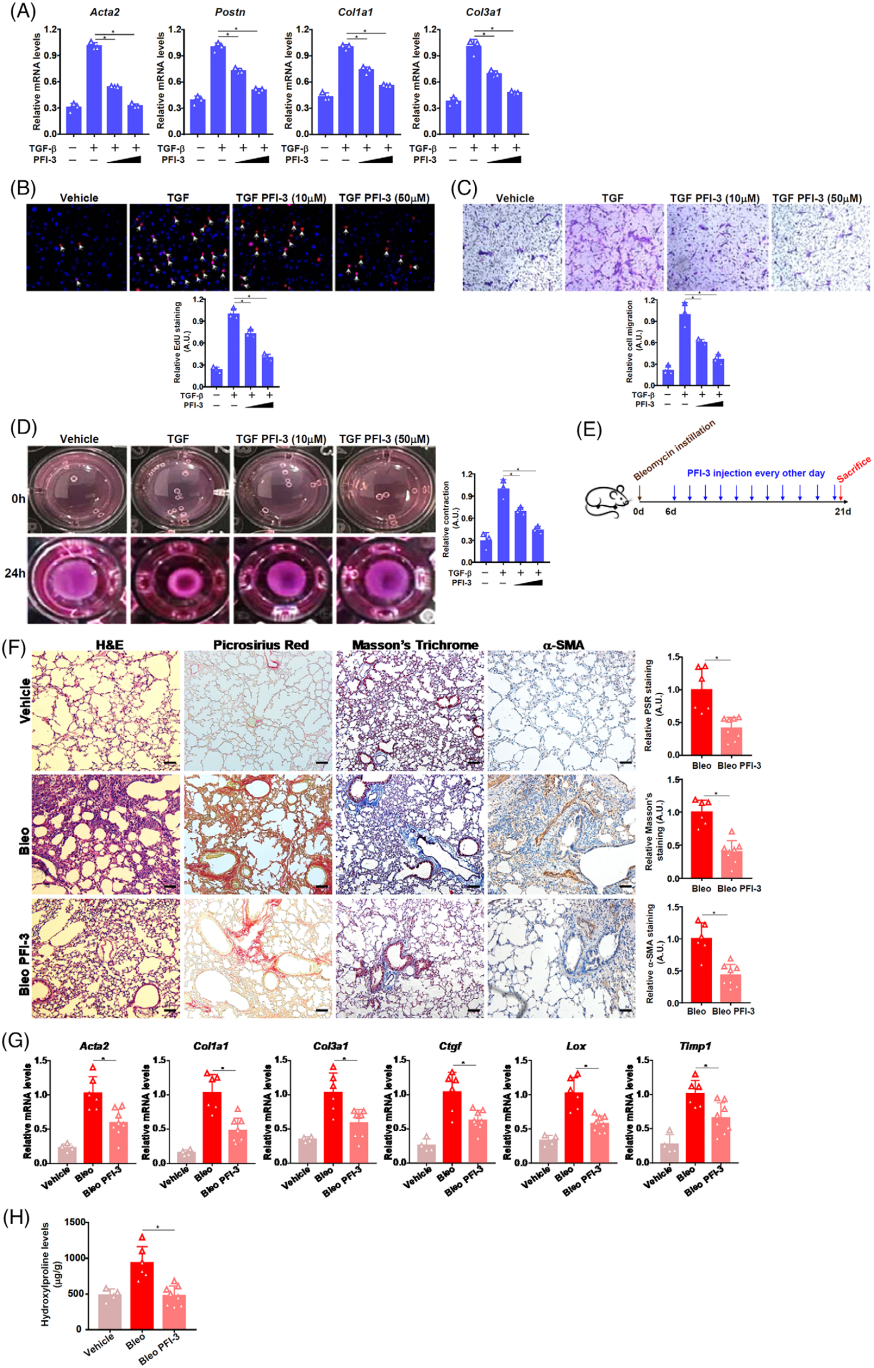
Brahma‐related gene 1 (BRG1) inhibition attenuates fibroblast‐myofibroblast transition in vitro and pulmonary fibrosis in mice. (A–D) Pulmonary fibroblasts were treated with transforming growth factor‐β (TGF‐β) (5 ng/mL) in the presence or absence of PFI‐3 for 24 h. Myofibroblast marker genes were examined by quantitative polymerase chain reaction (qPCR) (A). EdU incorporation (B). Boyden transwell (C). Collagen contraction assay (D). *N* = 3 biological replicates. Data are expressed as mean ± S.D. *, *p*<0.05, one‐way analysis of variance (ANOVA) with post‐hoc Scheff´e. (E–H) C57/BL6 mice were subjected to bleomycin instillation to induce pulmonary fibrosis followed by intervention with PFI‐3 (30µg/kg). Scheme of experimental protocol (E). Paraffin sections were stained with Picrosirius Red and Masson's trichrome (F). Pro‐fibrogenic genes were examined by qPCR (G). Hydroxyproline levels (H).

When primary murine fibroblasts were treated with TGF‐β in the presence or absence of PFI‐3 followed by RNA sequencing (RNA‐seq) (Figure S6A), more than a thousand differentially expressed genes were identified (Figure S6B). Further analyses indicated that PFI‐3 primarily altered the expression of genes related to fibroblast‐myofibroblast transition by inhibiting pro‐fibrogenic transcription factors including nuclear factor kappa B (NF‐κB), TEAD, SRF, and AP‐1 (Figure S6C–F). QPCR examination verified that *Ccl7*, *Adamts5*, *Itga8*, *Dmpk*, and *Gas6*, all ranked among the top 10 differentially expressed genes, were up‐regulated by TGF‐β treatment but down‐regulated by PFI‐3 treatment (Figure S6G). We focused on CCL7 for the remainder of the study because CCL7 appeared to be altered most significantly by TGF‐β and PFI‐3. CCL7 levels were robustly up‐regulated in pulmonary fibroblasts isolated from the mice induced to develop pulmonary fibrosis (Figure S7A,B). This observation was consistent with the published studies in which CCL7 expression was shown to increase in the lung tissues of bleomycin‐administered mice (Figure S8, bulk RNA‐seq). Additionally, it was noted that CCL7 levels were higher in the IPF lung tissues than in the normal lung tissues (Figure S7C) and in pulmonary fibroblasts from IPF patients (Figure S9 single‐cell RNA‐seq). Importantly, CCL7 levels were found to be positively correlated with those of myofibroblast markers (Figures S7D and S10). Single‐cell RNA‐seq also indicated that BRG1 levels were selectively elevated in lipofibroblasts and myofibroblasts in the lungs (Figure S11). TGF‐β treatment up‐regulated CCL7 expression whereas BRG1 deletion dampened CCL7 induction (Figure S7E,F). ChIP assays detected a stronger association of BRG1 with the CCL7 proximal promoter in both lung tissues from the bleomycin‐injected mice (Figure S7G) and pulmonary fibroblasts treated with TGF‐β (Figure S7H).

Treatment with recombinant CCL7 promoted (Figures S12 and S13) whereas CCL7 knockdown blocked (Figures S14 and S15) fibroblast‐myofibroblast transition in vitro. ShRNA targeting CCL7 was placed under the control of a *Postn* promoter, packaged into AAV6, and injected into C57/BL6 mice followed by bleomycin instillation (Figure 3A). CCL7 knockdown in mice significantly attenuated pulmonary fibrosis (Figure 3B‐‐D) without altering pulmonary inflammation (Figure S16). Next, C57/BL6 mice were given bleomycin followed by CCL7 depletion with a CCL7‐neutralizing antibody (Figure 3E). CCL7 blockade similarly mitigated pulmonary fibrosis (Figure 3F‐‐H). Again, pulmonary inflammation was largely unaltered by CCL7 neutralization (Figure S17).

**FIGURE 3:**
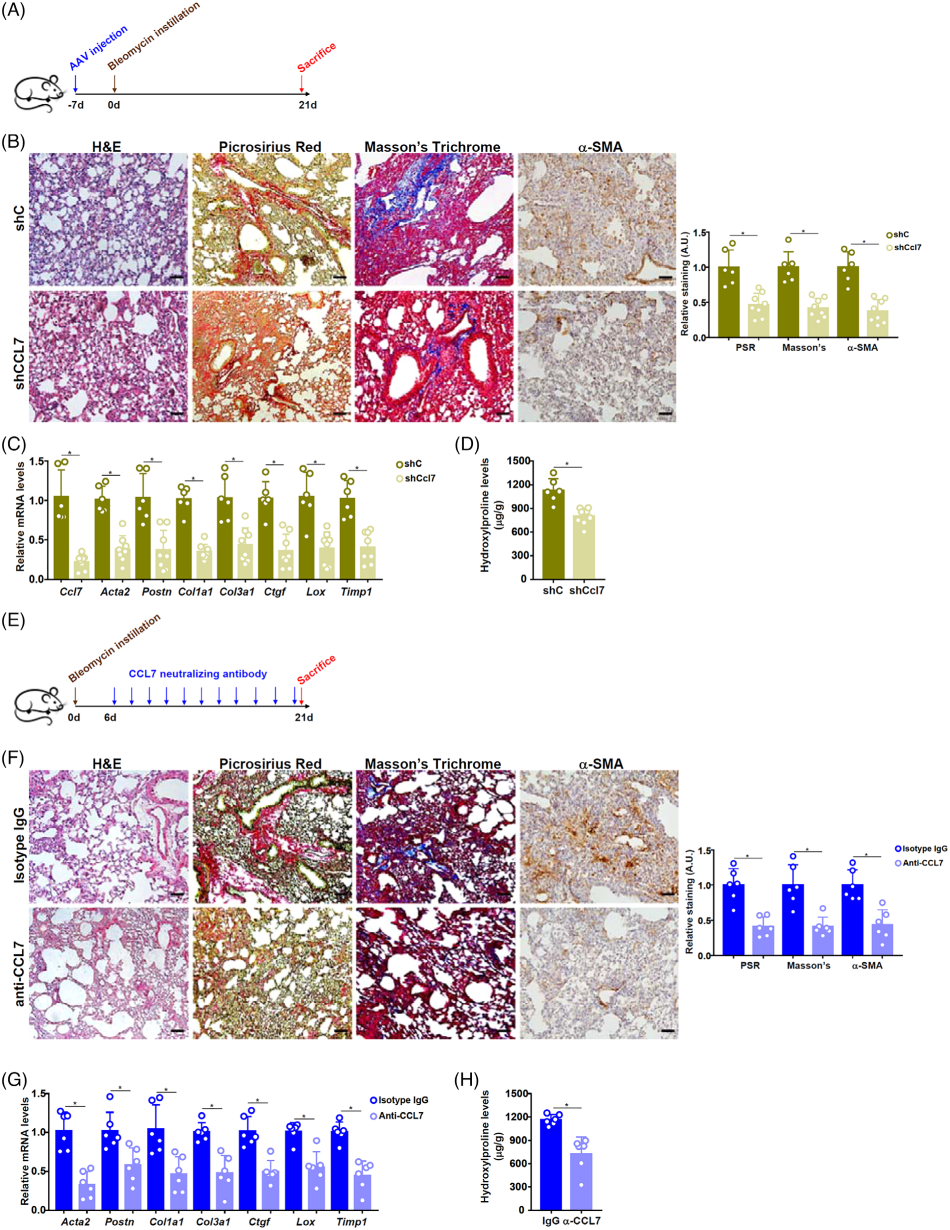
CCL7 targeting alleviates pulmonary fibrosis in mice. (A–D) C57/BL6 were injected with AAV6 carrying short‐hairpin RNA (shRNA) targeting CCL7 (shCcl7) or control shRNA (shC) followed by bleomycin distillation to induce pulmonary fibrosis. Scheme of protocol (A). Paraffin sections were stained with Picrosirius Red and Masson's trichrome (B). Pro‐fibrogenic genes were examined by quantitative polymerase chain reaction (qPCR) (C). Hydroxyproline levels (D). (E–H) C57/BL6 mice were subjected to bleomycin instillation to induce pulmonary fibrosis followed by intervention with a CCL7‐neutralizing antibody (500 µg/kg). Scheme of protocol (E). Paraffin sections were stained with Picrosirius Red and Masson's trichrome (F). Pro‐fibrogenic genes were examined by qPCR (G). Hydroxyproline levels (H).

CCL7 knockdown in IPF fibroblasts altered cellular transcriptome leading to 3000+ genes to be differentially expressed (Figure 4A,B). Further analysis showed that CCL7 primarily influenced the expression of genes involved in ECM remodelling through canonical pro‐fibrogenic signalling pathways (Figure 4C‐‐E), which was confirmed by reporter assay (Figure 4F). Immunofluorescence staining showed that CCL7 depletion reduced the nuclear localization of STAT6/SMAD3/NF‐κB (Figure 4G). As a result, occupancies of STAT6/SMAD3/NF‐κB on the *POSTN* promoter and the *COL1A2* promoter were collectively down‐regulated (Figure 4H). In contrast, rCCL7 treatment stimulated STAT6/SMAD3/NF‐κB activities by promoting nuclear accumulation and promoter recruitment (Figure S18).

**FIGURE 4:**
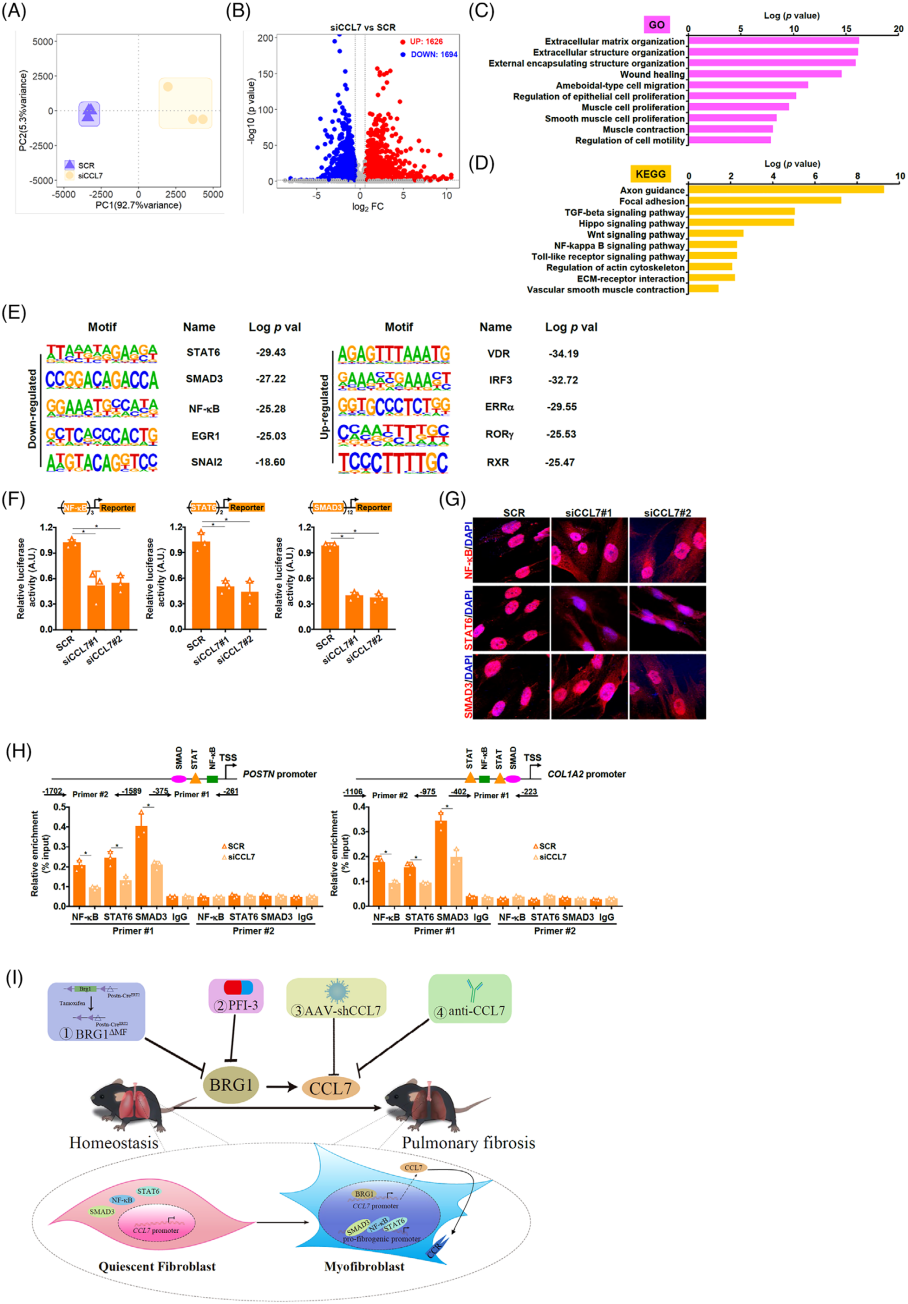
CCL7 contributes to fibroblast‐myofibroblast transition by modulating STAT6/SMAD3/NF‐κB activity. (A–E) Pulmonary fibroblasts from idiopathic pulmonary fibrosis (IPF) patients were transfected with indicated small‐interfering RNAs (siRNAs) followed by RNA sequencing (RNA‐seq). Principal component analysis (PCA) plot (A). Volcano plot (B). Gene ontology (GO) analysis (C). Kyoto Encyclopedia of Genes and Genomes (KEGG) analysis (D). Hypergeometric Optimization of Motif EnRichment (HOMER) analysis (E). (F) Reporter constructs were transfected into IPF cells along with indicated siRNAs. Luciferase activities were normalized by protein concentration and green fluorescent protein (GFP) fluorescence. (G) IPF cells were transfected with indicated siRNAs. Protein subcellular localization was evaluated by immunofluorescence staining. (H) IPF cells were transfected with indicated siRNAs. ChIP assays were performed with anti‐NF‐κB, anti‐STAT6, anti‐SMAD3, or immunoglobulin G (IgG). (I) A schematic model.

In summary, we describe here the essential role of the chromatin‐remodelling protein BRG1 in regulating myofibroblasts in the lungs. More importantly, our data highlight the translational potential of the BRG1‐CCL7 axis by providing proof‐of‐concept evidence that targeting BRG1 or CCL7 could be considered a reasonable approach for the intervention of pulmonary fibrosis (Figure 4I).

## AUTHOR CONTRIBUTIONS

Ming Kong and Yong Xu conceived the project; Teng Wu, Bingshu Wang, Xianhua Gui, Ruiqi Liu, and Dong Wei designed experiments, performed experiments, collected data, and analyzed data; all authors contributed to manuscript drafting and editing; Yong Xu, Nan Li and Shaojiang Zheng secured funding and provided supervision.

## CONFLICT OF INTEREST STATEMENT

The authors declare no conflict of interest.

## Supporting information

Supporting Information

## Data Availability

The data that support the findings of this study are available upon reasonable request.
