# Supplementary material for: Targeting the chromatin remodelling protein Brahma‐related gene 1 for intervention of pulmonary fibrosis
Source: Clin Transl Med. 2024 Aug 21;14(8):e1775. doi: 10.1002/ctm2.1775 (PMC11337535; doi:10.1002/ctm2.1775)
Supplement: Supplementary file 1 — Supporting Information [file CTM2-14-e1775-s001.docx]

**Wu T et al: Targeting the chromatin remodeling protein BRG1 for intervention of pulmonary fibrosis**

**Online supplementary material**

**Supplementary Methods: 1**

**Supplementary Discussion: 1**

**Supplementary figures: 18**

**Supplementary tables: 3**

**Methods**

*Animals*

All animal experiments were reviewed and approved by the Ethics Committee on Humane Treatment of Laboratory Animals of Nanjing Medical University and were performed in accordance with the ethical standards laid down in the 1964 Declaration of Helsinki and its later amendments. *Smarca4*^f/f^ mice ([1](#_ENREF_1)) and *Postn*-Cre^ERT2^ mice ([2](#_ENREF_2)) have been described previously. To induce pulmonary fibrosis, male 8-wk-old mice were anesthetized with isoflurane and treated with bleomycin (0.1 U/100 μL saline/20 g mouse) or equivalent volume of saline via intratracheal instillation. Animals were killed at 7-21 days after bleomycin instillation. To induce Cre expression in the *Postn*-Cre^ERT2^ mice, tamoxifen was injected peritoneally (50 mg/kg) for 5 consecutive days followed by maintaining the mice on a TAX-containing diet (Cat#: TD.130855, Inotiv) until the day when the mice were sacrificed. In certain experiments, the mice were injected peritoneally PFI-3 (Cat#: S7315, Selleck) at a dose of 50 mg/kg. In certain experiments, the mice were injected with a CCL7-neutralizing antibody (Cat#AF-456, Roche, 500μg/kg) or an isotype IgG every other day. In certain experiments, recombinant AAV6 carrying CCL7 shRNA (AGAGGAAUCUCAAGAGCUATT) downstream of the *Postn* promoter ([3](#_ENREF_3)). The AAV was injected into C57/B6 mice intravenously (i.v.) at a dose of 1×10^11^ vg.

*Cell culture, plasmids, and transient transfection*

Primary murine pulmonary fibroblasts were isolated from adult C57/BL6 mice and cultured as previously described ([4](#_ENREF_4)). Primary pulmonary fibroblasts from IPF patients were isolated and maintained as previously described ([5](#_ENREF_5)). Small interfering RNAs were purchased from Dharmacon. SMAD reporter ([6](#_ENREF_6)), STAT6 reporter ([7](#_ENREF_7)), and NF-κB reporter ([8](#_ENREF_8)) constructs have been previously described. Transient transfections were performed with Lipofectamine 2000. Luciferase activities were assayed 24-48 hours after transfection using a luciferase reporter assay system (Promega) as previously described ([9](#_ENREF_9)).

*RNA Isolation and Real-time PCR*

RNA was extracted with the RNeasy RNA isolation kit (Qiagen) as previously described ([10](#_ENREF_10)). Reverse transcriptase reactions were performed using a SuperScript First-strand Synthesis System (Invitrogen). Real-time PCR reactions were performed on an ABI Prism 7500 system. The primers are list in the supplementary Table I. Ct values of target genes were normalized to the Ct values of house-keeping control gene (18s, 5’-CGCGGTTCTATTTTGTTGGT-3’ and 5’-TCGTCTTCGAAACTCCGACT-3’ for both human and mouse genes) using the ΔΔCt method and expressed as relative mRNA expression levels compared to the control group which is arbitrarily set as 1.

*Protein extraction and Western blotting*

Whole cell lysates were obtained by re-suspending cell pellets in RIPA buffer (50 mM Tris pH7.4, 150 mM NaCl, 1% Triton X-100) with freshly added protease inhibitor (Roche). Western blot analyses were performed with the antibodies listed in supplementary Table II.

*Chromatin Immunoprecipitation (ChIP)*

Chromatin immunoprecipitation (ChIP) assays were performed essentially as described before ([11](#_ENREF_11), [12](#_ENREF_12)). Chromatin was cross-linked with 1% formaldehyde for 8 min at room temperature and then sequentially washed with ice-cold phosphate-buffered saline, Solution I (10 mM HEPES, pH 7.5, 10 mM EDTA, 0.5 mM EGTA, 0.75% Triton X-100), and Solution II (10 mM HEPES, pH 7.5, 200 mMNaCl, 1 mM EDTA, 0.5 mM EGTA). Cells were incubated in lysis buffer (150 mMNaCl, 25 mMTris pH 7.5, 1% Triton X-100, 0.1% SDS, 0.5% deoxycholate) supplemented with a protease inhibitor tablet. DNA was fragmented into 500 bp pieces using a Branson 250 sonicator. Aliquots of lysates containing 100 μg of protein were used for each immunoprecipitation reaction with the indicated antibodies followed by adsorption ontoprotein A/G PLUS-agarose beads (Santa Cruz Biotechnology). Precipitated DNA‒protein complexes were washed sequentially with RIPA buffer (50 mMTris, pH 8.0, 150 mMNaCl, 0.1% SDS, 0.5% deoxycholate, 1% Nonidet P-40, 1 mM EDTA), high salt buffer (50 mMTris, pH 8.0, 500 mMNaCl, 0.1% SDS, 0.5% deoxycholate, 1% Nonidet P-40, 1 mM EDTA), LiCl buffer (50 mMTris, pH 8.0, 250 mMLiCl, 0.1% SDS, 0.5% deoxycholate, 1% Nonidet P-40, 1 mM EDTA), and TE buffer (10 mMTris, 1 mM EDTA pH 8.0). DNA‒protein cross-linking was reversed by heating the samples to 65 °C overnight. Proteins were digested with proteinase K (Sigma), and DNA was phenol/chloroform-extracted and precipitated by 100% ethanol. Precipitated genomic DNA. A total of 10% of the starting material was also included as the input.

*Boyden chamber trans-well assay*

The cells were trypsinized and seeded into Boyden chambers (PET track-etched, 8-μm pores, 24-well format; Becton Dickinson) in serum-free DMEM medium. Complete culture medium containing 10% FBS was added to the lower chamber. The cells migrating from the upper chamber were fixed with 4% paraformaldehyde, stained with 0.1% crystal violet, and counted under a microscope. Cell numbers from 5 random fields were counted in each well.

*Collagen contraction assay*

The cells were trypsinized, mixed with 4x the volume of Collagen Gel Working Solution (Corning) and incubated for 1 hr at 37ºC. 4. After collagen polymerization, 1.0 mL of culture medium was added atop. The collagen gel size change was measured 24h later and quantified with Image Pro Plus.

*EdU incorporation assay*

5-ethynyl-2’-deoxyuridine (EdU) incorporation assay was performed in triplicate wells with a commercially available kit (Thermo Fisher) as previously described ([12](#_ENREF_12)). Briefly, the EdU solution was diluted with the culture media and added to the cells for an incubation period of 2h at 37^o^C. After several washes with 1XPBS, the cells were then fixed with 4% formaldehyde and stained with Alexa Fluor™ 488. The nucleus was counter-stained with DAPI. The images were visualized by fluorescence microscopy and analyzed with Image-Pro Plus (Media Cybernetics). For each group, at least six different fields were randomly chosen and the positively stained cells were counted and divided by the number of total cells. The data are expressed as relative EdU staining compared to the control group arbitrarily set as 1.

*Immunofluorescence staining*

Paraffin sections were blocked with 5% BSA and incubated with the indicated primary antibodies overnight. After several washes with PBS, the slides were incubated with FITC-labeled secondary antibodies (Jackson) for 30 min. DAPI (Sigma) was added and incubated for 5 min prior to observation. Immunofluorescence was visualized undera confocal microscope (LSM 710, Zeiss).

*Enzyme-Linked Immunosorbent Assay*

Secreted CCL7 levels were examined by ELISA as previously described using commercially available kits (Abcam, ab205571) according to vendor’s recommendations. Data were normalized by cell number (for supernatant collected from primary cell culture).

*Histology*

Histological analyses were performed essentially as described before ([12-14](#_ENREF_12)). Pictures were taken using an Olympus IX-70 microscope. Quantifications were performed with ImageJ. For each mouse, at least three slides were stained, and at least five different fields were analyzed ineach slide.

*Human lung tissue specimens*

Normal control lungs were obtained with approval by the Ethics Committee on Human Tissue Collection and Processing of Wuxi People’s Hospital, following rejection as candidate donors for transplant. IPF lung tissues were obtained with approval by the Ethics Committee on Human Tissue Collection and Processing of Wuxi People’s Hospital during transplantation surgery. Subject information is provided in supplementary Table III.

*RNA Sequencing and Data Analysis*

RNA-seq was performed as previously described ([15-17](#_ENREF_15)). Total RNA was extracted using TRIzol reagent according to the manufacturer’s protocol. RNA purity and quantity were evaluated using a NanoDrop 2000 spectrophotometer (Thermo Scientific, USA). RNA integrity was assessed using the Agilent 2100 Bioanalyzer (Agilent Technologies, Santa Clara, CA, USA). Then, the libraries were constructed using the TruSeq Stranded mRNA LT Sample Prep Kit (Illumina, San Diego, CA, USA) according to the manufacturer's instructions and were sequenced on an Illumina HiSeq X Ten platform, and 150 bp paired-end reads were generated. Raw data (raw reads) in fastq format were first processed using Trimmomatic, and low-quality reads were removed to leave only clean reads. The clean reads were mapped to the mouse genome (Mus_musculus.GRCm38.99) using HISAT2. The FPKM of each gene was calculated using Cufflinks, and the read counts of each gene were obtained by HTSeqcount. Differential expression analysis was performed using the DESeq (2012) R package. A P value < 0.05 and a fold change > 2 or < 0.5 was set as the threshold for significantly differential expression. Hierarchical cluster analysis of differentially expressed genes (DEGs) was performed to demonstrate the expression pattern of genes in different groups and samples. GO enrichment and KEGG pathway enrichment analyses of DEGs were performed using R based on the hypergeometric distribution. Analysis of transcription factor binding motifs at the promoters of DEGs was performed using HOMER. The configureHomer.pl script was used to download the mouse promoter reference for the analysis. The findMotifs.pl script was used to perform motif analysis in the promoter regions from −2000 to +100 bp relative to TSS (transcription start site). The motif instances were generated according to the matching sequences as well as the position-weight matrix.

*Statistical analysis*

For comparison between two groups, two-tailed t-test was performed. For comparison among three or more groups, one-way ANOVA or two-way ANOVA with post-hoc Turkey analyses were performed using an SPSS package. The assumptions of normality were checked using Shapiro-Wilks test and equal variance was checked using Levene's test; both were satisfied. *p* values smaller than .05 were considered statistically significant (*). All *in vitro* experiments were repeated at least three times and three replicates were estimated to provide 80% power.

**Discussion**

ECM-producing myofibroblasts contributes to pulmonary fibrosis by mediating interstitial remodeling. Here we detail a novel pathway wherein the chromatin remodeling protein BRG1 regulates pulmonary fibrosis by modulating myofibroblast phenotype (Fig.8I). BRG1 belongs to the SWI/SNI family of chromatin remodeling proteins providing the ATPase activity to mobilize nucleosomes ([18](#_ENREF_18)). BRG1 plays a versatile role in pathogenesis of a wide range of human diseases. For instance, *SMARCA4* (the BRG1-encoding gene) has been shown to be frequently mutated in human cancers including lung cancer and both a pro-oncogenic and an anti-oncogenic role have been proposed for BRG1 ([19-21](#_ENREF_19)). Previously, it has been reported that BRG1 can contribute to liver fibrosis ([22](#_ENREF_22)) and renal fibrosis ([23](#_ENREF_23)). Consistent with previous studies performed in keratinocytes ([24](#_ENREF_24), [25](#_ENREF_25)), we show here that BRG1 deletion significantly blockaded transition of pulmonary fibroblasts into myofibroblasts and mitigated pulmonary fibrosis *in vivo*. More importantly, BRG1 inhibition by PFI-3 exhibited therapeutic potential in bleomycin-induced pulmonary fibrosis. Of intrigue, whereas BRG1 deletion in myofibroblasts diminished fibrogenesis in the lungs without altering the inflammatory response, PFI-3 administration led to simultaneous lightened pulmonary and inflammation suggesting that BRG1 might play compartmentalized roles in regulating pulmonary pathology. Indeed, mounting evidence portrays BRG1 as a pivotal orchestrator of immune response by influencing the phenotype of T cells ([26](#_ENREF_26)), B cells ([27](#_ENREF_27)), macrophages ([28](#_ENREF_28)), and granulocytes ([29](#_ENREF_29)). Because infiltration of immune cells and the ensuing inflammatory response are observed during and considered key to the pathogenesis of pulmonary fibrosis ([30](#_ENREF_30)), it is likely that BRG1 could contribute to pulmonary fibrosis by modulating immunity in parallel to programming fibroblast-myofibroblast transition. It would be worthwhile to examine whether BRG1 deletion in one or more of these immune cell lineages would phenocopy the effects of PFI-3 *in vivo*. Alternatively, PFI-3 targets all the bromodomain-containing proteins, which include 46 members in humans ([31](#_ENREF_31)). It is reasonable to postulate that one or more of these proteins, in addition to BRG1, may possess anti-fibrogenic and/or anti-inflammatory properties to be implicated into pulmonary fibrosis. Clearly more investigations are warranted to determine the specificity of PFI-3 so that its potential usage as a real-world therapeutic approach against pulmonary fibrosis can be justified.

One of the most interesting findings in this report is that CCL7, directly activated by BRG1 in fibroblasts, possesses a previously unrecognized pro-fibrogenic role promoting fibroblast-myofibroblast transition and pulmonary fibrosis. Members of the C-C motif chemokine ligand super-family, including CCL7, are emphatically assigned the role of steering cell trafficking. However, recent studies have uncovered a range of uncanonical functions for the CCL proteins. Clement *et al* have shown that adipocyte-derived CCL2 stimulates *de novo* lipogenesis and consequently lipid accumulation in hepatocytes ([32](#_ENREF_32)). Fan *et al* have similarly found that an auto-regulatory loop fueled by hepatocyte-derived CCL11 promotes hepatic lipid accumulation ([33](#_ENREF_33)). More relevant to the present study, Puxeddu *et al* have shown that pulmonary fibroblasts exposed to CCL11 display a myofibroblast-like phenotype *in vitro* ([34](#_ENREF_34)). Despite our finding that adds to an ever-expanding spectrum of pathobiological functions for CCL proteins, it remains unclear whether the pro-fibrogenic effect observed for CCL7 in pulmonary fibrosis can be extrapolated to other scenarios (e.g., cardiac fibrosis or renal fibrosis). Our group has recently demonstrated that myofibroblast-specific deletion of CCL11 attenuates liver fibrosis in mice likely owing to dampened Notch signaling ([35](#_ENREF_35)). Of note, CCL7 and CCL11 share two receptors, CCR3 and CCR5, indicating that there might be a redundancy or synergism between these two molecules during organ fibrosis.

It is worth noting that our transcriptomic screening revealed more hits than CCL7 that could potentially mediate the pro-fibrogenic effect of BRG1, some of which were further validated by qPCR (Fig.4G). Growth arrested-specific gene 6 (Gas6), a secreted protein that signals through the tyrosine kinase receptors Tyro3, Axl and Mertk, has been used a biomarker for pulmonary fibrosis ([36](#_ENREF_36)). Interestingly, Gas6 deletion appears to attenuate pulmonary fibrosis in models of fungal allergic airway disease, smoking, and pneumoconiosis ([37-39](#_ENREF_37)) although it remains undecided how the anti-fibrotic effect of Gas6 deficiency was achieved since all the studies used a global rather than conditional Gas6 knockout strain. Integrin α8 (Itga8) has recently been used to define a specific fibroblast sub-population in IPF patients that resides in the elastic fiber-rich connective tissue of the lungs ([40](#_ENREF_40)). Curiously, Itga8 deletion from PDGRFβ^+^ cells failed to influence pulmonary fibrosis at day 21 following bleomycin instillation suggesting that Itga8 may exert its effect in alternative lineages or at different stages of pulmonary fibrosis ([41](#_ENREF_41)). We propose that BRG1 may program fibroblast-myofibroblast transition by simultaneously regulating a network of target genes, which in turn act concertedly to compel a phenotypic switch. Further studies are warranted to verify this model by validating each individual BRG1 targets in FMyT and pulmonary fibrosis.

In summary, we describe here an essential role for the chromatin remodeling protein BRG1 in regulating myofibroblasts in the lungs and, more importantly, provide proof-of-concept evidence that targeting BRG1 could be considered as a reasonable approach for the intervention of pulmonary fibrosis. However, there are major limitations regarding the conclusiveness of our study. For instance, he relationship between BRG1 and CCL7 in promoting FMyT and pulmonary fibrosis is largely correlative but not causal. Additional experiments should be performed to clarify whether the profibrotic effect of BRG1 is dependent on CCL7. Before an all-out effort is invested on translating these findings into therapies, it remains to be answered how BRG1 influences epigenetic landscape and chromatin accessibility to coordinate the binding of transcription factors and basal transcription machinery during fibroblast-myofibroblast transition. Future studies should focus on the solving these lingering issues to generate safe and effective therapeutic strategies for the treatment of pulmonary fibrosis.

**Fig.S1:** (**A, B**) C57/BL6 mice were subjected to intratracheal instillation with bleomycin or saline. The mice were sacrificed at indicated time points for isolation of pulmonary fibroblasts. BRG1 expression was examined by qPCR and Western blotting. N=5 mice for each group. Data are expressed as mean±S.D. *, *p*＜0.05, one-way ANOVA with post-hoc Scheff´e. (**C, D**) Primary murine pulmonary fibroblasts and MRC5 cells were treated with TGF-β (5ng/ml). Cells were harvested at indicated time points and BRG1 expression was examined by qPCR and Western blotting. N=3 biological replicates. Data are expressed as mean±S.D. *, *p*＜0.05, one-way ANOVA with post-hoc Scheff´e. (**E, F**) BRG1 expression in lungs from IPF patients and healthy donors was examined by qPCR. N=7 cases for each group. Data are expressed as mean±S.D. *, *p*＜0.05, two-tailed student’s test. Linear regression was performed using Graphpad.

**Fig.S2:** (**A-D**) Pulmonary fibroblasts isolated from IPF patients were transfected with indicated siRNAs. Myofibroblast marker genes were examined by qPCR (A). EdU incorporation (B). Boyden transwell (C). Collagen contraction assay (D).

**Fig.S3:** Myofibroblast conditional BRG1 deletion mice (BRG1^ΔMF^) and wild type (WT) control mice were subjected to bleomycin instillation to induce pulmonary fibrosis. The mice were sacrificed at day 7 post-treatment. (**A**) Paraffin sections were stained with anti-F4/80 or anti-CD45. (**B**) Pro-inflammatory genes were examined by qPCR.

**Fig.S4:** (**A-D**) Pulmonary fibroblasts isolated from IPF patients were treated with PFI-3 (10μM and 50μM) for 24h. Myofibroblast marker genes were examined by qPCR (A). EdU incorporation (B). Boyden transwell (C). Collagen contraction assay (D).

**Fig.S5:** C57/BL6 mice were subjected to bleomycin instillation to induce pulmonary fibrosis followed by intervention with PFI-3 (30μg/kg). The mice were sacrificed at day 7 post-treatment. (**A**) Paraffin sections were stained with anti-F4/80 or anti-CD45. (**B**) Pro-inflammatory genes were examined by qPCR.

**Figure S6:** (**A-F**) Primary murine pulmonary fibroblasts were treated with TGF-β (5ng/ml) in the presence or absence of PFI-3 (50μM) for 24h. RNA-seq was performed as described in Methods. PCA plot (A). Volcano plot (B). GO analysis (C). KEGG analysis (D). HOMER analysis (E). Heatmap of differentially expressed genes (F). (**G**) Validation of novel BRG1 targets by qPCR.

**Figure S7:** (**A, B**) C57/BL6 mice were subjected to intratracheal instillation with bleomycin or saline. The mice were sacrificed at indicated time points for isolation of pulmonary fibroblasts. CCL7 expression was examined by qPCR and ELISA. N=5 ice for each group. Data are expressed as mean±S.D. *, *p*＜0.05, one-way ANOVA with post-hoc Scheff´e. (**C, D**) CCL7 expression in lungs from IPF patients and healthy donors was examined by qPCR. N=7 cases for each group. Data are expressed as mean±S.D. *, *p*＜0.05, two-tailed student’s test. Linear regression was performed using Graphpad. (**E, F**) Pulmonary fibroblasts were isolated from BRG1^f/f^ mice and transduced with indicated adenovirus followed by treatment with TGF-β (5ng/ml). The cells were harvested at indicated time points and CCL7 expression was examined by qPCR and ELISA. N=3 biological replicates. Data are expressed as mean±S.D. *, *p*＜0.05, one-way ANOVA with post-hoc Scheff´e. (**G**) C57/BL6 mice were subjected to intratracheal instillation with bleomycin and sacrificed at indicated time points. ChIP assays were performed with anti-BRG1 or IgG using lung tissue homogenates. N=3 mice for each group. Data are expressed as mean±S.D. *, *p*＜0.05, one-way ANOVA with post-hoc Scheff´e. (**H**) Primary murine pulmonary fibroblasts were treated with TGF-β (5ng/ml) and harvested at indicated time points. ChIP assays were performed with anti-BRG1 or IgG using cell lysates. N=3 biological replicates. Data are expressed as mean±S.D. *, *p*＜0.05, one-way ANOVA with post-hoc Scheff´e.

**Fig.S8:** CCL7 expression in the lung tissues of mice subjected to bleomycin instillation was examined by RNA-seq. Data were exported from publicly deposited datasets and re-analyzed.

**Fig.S9:** CCL7 expression in pulmonary fibroblasts from IPF patients or healthy individuals was examined by single-cell RNA-seq. Data were exported from publicly deposited datasets and re-analyzed.

**Fig.S10:** Correlation between CCL7 expression and that of myofibroblast markers in pulmonary fibroblasts from IPF patients was performed with Graphpad. Data were exported from publicly deposited single-cell RNA-seq datasets and re-analyzed.

**Fig.S11:** BRG1 expression in pulmonary fibroblasts from IPF patients or healthy individuals was examined by single-cell RNA-seq. Data were exported from publicly deposited datasets and re-analyzed.

**Figure S12:** (**A-D**) Primary murine pulmonary fibroblasts were exposed to different doses of recombinant CCL7 for 24h. Myofibroblast marker genes were examined by qPCR (A). EdU incorporation (B). Boyden transwell (C). Collagen contraction assay (D).

**Fig.S13:** (**A-D**) MRC5 cells were exposed to different doses of recombinant CCL7 for 24h. Myofibroblast marker genes were examined by qPCR (A). EdU incorporation (B). Boyden transwell (C). Collagen contraction assay (D).

**Figure S14:** (**A-D**) Pulmonary fibroblasts from IPF patients were transfected with indicated siRNAs. Myofibroblast marker genes were examined by qPCR (A). EdU incorporation (B). Boyden transwell (C). Collagen contraction assay (D).

**Fig.S15:** (**A-D**) Primary murine pulmonary fibroblasts were transfected with indicated siRNAs followed by treatment with TGF-β (5ng/ml) for 24h. Myofibroblast marker genes were examined by qPCR (A). EdU incorporation (B). Boyden transwell (C). Collagen contraction assay (D).

**Fig.S16:** C57/BL6 were injected with AAV6 carrying shRNA targeting CCL7 (shCcl7) or control shRNA (shC) followed by bleomycin distillation to induce pulmonary fibrosis. The mice were sacrificed at day 7 post-treatment. (**A**) Paraffin sections were stained with anti-F4/80 or anti-CD45. (**B**) Pro-inflammatory genes were examined by qPCR.

**Fig.S17:** C57/BL6 were injected with a CCL7-neutralizing antibody or isotype IgG followed by bleomycin distillation to induce pulmonary fibrosis. The mice were sacrificed at day 7 post-treatment. (**A**) Paraffin sections were stained with anti-F4/80 or anti-CD45. (**B**) Pro-inflammatory genes were examined by qPCR.

**Fig.S18:** (**A**) Different reporter constructs were transfected into MRC5 cells followed by treatment with rCCL7 (50ng/ml) for 24h. Luciferase activities were normalized by protein concentration and GFP fluorescence. (**B**) MRC5 cells were treated with or without rCCL7 (50ng/ml) for 6h. Protein subcellular localization was evaluated by immunofluorescence staining. (**C**) MRC5 cells were treated with or without rCCL7 (50ng/ml) for 24h. ChIP assays were performed with anti-NF-κB, anti-STAT6, anti-SMAD3, or IgG.

**Table I: QPCR Primers Sequences**

| **Gene** | | **Forward primer** | | **Reverse primer** | |
| --- | --- | --- | --- | --- | --- |
| Mouse *Brg1* | | GAGCCAGAACGAGAAGTACCG | | CCTCAAGACGAGCAATTTCATCA | |
| Mouse *Col1a1* | | GCTCCTCTTAGGGGCCACT | | ATTGGGGACCCTTAGGCCAT | |
| Mouse *Col3a1* | | CTGTAACATGGAAACTGGGGAAA | | CCATAGCTGAACTGAAAACCACC | |
| Mouse *Acta2* | | CCCAGACATCAGGGAGTAATGG | | TCTATCGGATACTTCAGCGTCA | |
| Mouse *Col1a2* | | TCGTGCCTAGCAACATGCC | | TTTGTCAGAATACTGAGCAGCAA | |
| Mouse *Ctgf* | | GGCCTCTTCTGCGATTTCG | | GCAGCTTGACCCTTCTCGG | |
| Mouse *Postn* | | CCTGCCCTTATATGCTCTGCT | | AAACATGGTCAATAGGCATCACT | |
| Mouse *Lox* | | CAGCCACATAGATCGCATGGT | | GCCGTATCCAGGTCGGTTC | |
| Mouse *Ccl7* | | CCACATGCTGCTATGTCAAGA | | ACACCGACTACTGGTGATCCT | |
| Mouse *Timp1* | | CGAGACCACCTTATACCAGCG | | ATGACTGGGGTGTAGGCGTA | |
| Human *COL1A1* | | GAGGGCCAAGACGAAGACATC | | CAGATCACGTCATCGCACAAC | |
| Human *COL3A1* | | GGAGCTGGCTACTTCTCGC | | GGGAACATCCTCCTTCAACAG | |
| Human *ACTA2* | | AAAAGACAGCTACGTGGGTGA | | GCCATGTTCTATCGGGTACTTC | |
| Human *POSTN* | | CTCATAGTCGTATCAGGGGTCG | | ACACAGTCGTTTTCTGTCCAC | |
| Human *BRG1* | | GACCAGCACTCCCAAGGTTAC | | CTGGCCCGGAAGACATCTG | |
| Human *TIMP1* | | CTTCTGCAATTCCGACCTCGT | | ACGCTGGTATAAGGTGGTCTG | |
| Human *CCL7* | | CTTCAACTACCTGCTGCTACA | | GGGTTTTCTTGTCCAGGTGC | |
|  | |  | |  |  |

**Table II: Antibody information**

| **Antigen** | **Vendor (Cat#)** | **Application** |
| --- | --- | --- |
| BRG1 | Cell Signaling Tech (49360) | Western, ChIP |
| β-actin | Sigma (A2228) | Western |
| STAT6 | Proteintech (51073-1) | IF, ChIP |
| SMAD3 | Abcam (ab208182) | IF, ChIP |
| NF-κB | Cell Signaling Tech (8242) | IF, ChIP |
| F4/80 | Proteintech (28463-1) | IHC |
| CD45 | Proteintech (20103-1) | IHC |

**Table III: Human subject information**

| **ID** | **Gender** | **Age (yr)** | **FVC%** | **DLCO%** | **PO_2_ (mmHg)** | **PO_2_/FiO_2_** |
| --- | --- | --- | --- | --- | --- | --- |
| Healthy 1 | M | 57 | 81.2 | 79.8 | 87 | 414 |
| Healthy 2 | M | 62 | 88.1 | 83.1 | 85 | 405 |
| Healthy 3 | F | 67 | 80.3 | 82.2 | 79 | 376 |
| Healthy 4 | F | 61 | 79.8 | 85.4 | 82 | 390 |
| Healthy 5 | F | 75 | 80.1 | 81.1 | 84 | 400 |
| Healthy 6 | M | 54 | 86.1 | 80.1 | 89 | 429 |
| Healthy 7 | M | 62 | 82.9 | 83.2 | 78 | 371 |
| IPF 1 | F | 63 | 68.3 | 41.0 | 70 | 189 |
| IPF 2 | M | 65 | 67.9 | 50.3 | 79 | 213 |
| IPF 3 | M | 66 | 51.3 | 38.9 | 63 | 170 |
| IPF 4 | M | 62 | 64.1 | 30.8 | 59 | 159 |
| IPF 5 | M | 64 | 64.9 | 42.7 | 66 | 200 |
| IPF 6 | F | 63 | 55.6 | 51.6 | 73 | 197 |
| IPF 7 | M | 63 | 77.8 | 62.6 | 79 | 239 |

**References**

**1. Li N, H. L, Xue Y, Xu Z, Miao X, Guo Y, et al. Targetable Brg1-CXCL14 axis contributes to alcoholic liver injury by driving neutrophil trafficking. EMBO molecular medicine. 2023.**

**2. Kanisicak O, Khalil H, Ivey MJ, Karch J, Maliken BD, Correll RN, et al. Genetic lineage tracing defines myofibroblast origin and function in the injured heart. Nature communications. 2016;7:12260. Epub 2016/07/23.**

**3. Piras BA, Tian Y, Xu Y, Thomas NA, O'Connor DM, French BA. Systemic injection of AAV9 carrying a periostin promoter targets gene expression to a myofibroblast-like lineage in mouse hearts after reperfused myocardial infarction. Gene Ther. 2016;23(5):469-78. Epub 2016/03/02.**

**4. Baglole CJ, Reddy SY, Pollock SJ, Feldon SE, Sime PJ, Smith TJ, et al. Isolation and phenotypic characterization of lung fibroblasts. Methods in molecular medicine. 2005;117:115-27. Epub 2005/08/25.**

**5. Ramos C, Montano M, Garcia-Alvarez J, Ruiz V, Uhal BD, Selman M, et al. Fibroblasts from idiopathic pulmonary fibrosis and normal lungs differ in growth rate, apoptosis, and tissue inhibitor of metalloproteinases expression. American journal of respiratory cell and molecular biology. 2001;24(5):591-8. Epub 2001/05/15.**

**6. Yan X, Wu J, Jiang Q, Cheng H, Han JJ, Chen YG. CXXC5 suppresses hepatocellular carcinoma by promoting TGF-beta-induced cell cycle arrest and apoptosis. J Mol Cell Biol. 2018;10(1):48-59. Epub 2017/10/17.**

**7. Xiong X, Yang C, He WQ, Yu J, Xin Y, Zhang X, et al. Sirtuin 6 maintains epithelial STAT6 activity to support intestinal tuft cell development and type 2 immunity. Nature communications. 2022;13(1):5192. Epub 2022/09/04.**

**8. Yu L, Fang F, Dai X, Xu H, Qi X, Fang M, et al. MKL1 defines the H3K4Me3 landscape for NF-kappaB dependent inflammatory response. Scientific reports. 2017;7(1):191. Epub 2017/03/17.**

**9. Fan Z, Kong M, Dong W, Dong C, Miao X, Guo Y, et al. Trans-activation of eotaxin-1 by Brg1 contributes to liver regeneration. Cell death & disease. 2022;13(5):495. Epub 2022/05/26.**

**10. Wu T, Li N, Zhang Q, Liu R, Zhao H, Fan Z, et al. MKL1 fuels ROS-induced proliferation of vascular smooth muscle cells by modulating FOXM1 transcription. Redox biology. 2023;59:102586. Epub 2023/01/02.**

**11. Dong W, Zhu Y, Zhang Y, Fan Z, Zhang Z, Fan X, et al. BRG1 Links TLR4 Trans-Activation to LPS-Induced SREBP1a Expression and Liver Injury. Frontiers in cell and developmental biology. 2021;9:617073. Epub 2021/04/06.**

**12. Shao T, Xue Y, Fang M. Epigenetic Repression of Chloride Channel Accessory 2 Transcription in Cardiac Fibroblast: Implication in Cardiac Fibrosis. Frontiers in cell and developmental biology. 2021;9:771466. Epub 2021/12/07.**

**13. Cai J, Wang T, Zhou Y, Tang C, Liu Y, Dong Z. Phosphorylation by GSK-3beta increases the stability of SIRT6 to alleviate TGF-beta-induced fibrotic response in renal tubular cells. Life sciences. 2022;308:120914. Epub 2022/09/04.**

**14. Zhu B, Ni Y, Gong Y, Kang X, Guo H, Liu X, et al. Formononetin ameliorates ferroptosis-associated fibrosis in renal tubular epithelial cells and in mice with chronic kidney disease by suppressing the Smad3/ATF3/SLC7A11 signaling. Life Sci. 2022;315:121331. Epub 2023/01/01.**

**15. Wu X, Dong W, Kong M, Ren H, Wang J, Shang L, et al. Down-regulation of CXXC5 de-represses MYCL1 to promote hepatic stellate cell activation. Frontiers in cell and developmental biology. 2021;9:680344.**

**16. Liu S, Liu J, Wang Y, Deng L, Chen S, Wang X, et al. Differentially expressed genes induced by beta-caryophyllene in a rat model of cerebral ischemia-reperfusion injury. Life sciences. 2021;273:119293. Epub 2021/03/12.**

**17. Yao X, Zhu Z, Manandhar U, Liao H, Yu T, Wang Y, et al. RNA-seq reveal RNA binding protein GNL3 as a key mediator in the development of psoriasis vulgaris by regulating the IL23/IL17 axis. Life sciences. 2022;293:119902. Epub 2021/09/07.**

**18. Filippakopoulos P, Knapp S. Targeting bromodomains: epigenetic readers of lysine acetylation. Nature reviews Drug discovery. 2014;13(5):337-56. Epub 2014/04/23.**

**19. Mardinian K, Adashek JJ, Botta GP, Kato S, Kurzrock R. SMARCA4: Implications of an Altered Chromatin-Remodeling Gene for Cancer Development and Therapy. Molecular cancer therapeutics. 2021;20(12):2341-51. Epub 2021/10/14.**

**20. Wu Q, Lian JB, Stein JL, Stein GS, Nickerson JA, Imbalzano AN. The BRG1 ATPase of human SWI/SNF chromatin remodeling enzymes as a driver of cancer. Epigenomics. 2017;9(6):919-31. Epub 2017/05/20.**

**21. Navickas SM, Giles KA, Brettingham-Moore KH, Taberlay PC. The role of chromatin remodeler SMARCA4/BRG1 in brain cancers: a potential therapeutic target. Oncogene. 2023;42(31):2363-73. Epub 2023/07/12.**

**22. Zhu Y, Kang A, Kuai Y, Guo Y, Miao X, Zhu L, et al. The chromatin remodeling protein BRG1 regulates HSC-myofibroblast differentiation and liver fibrosis. Cell death & disease. 2023;14(12):826. Epub 2023/12/14.**

**23. Hong W, Zhu Y, Lin Y, Tang S, Chen J, Xu L, et al. The chromatin remodeling protein BRG1 mediates Ang II induced pro-fibrogenic response in renal fibroblasts. Life sciences. 2024;340:122320. Epub 2024/01/26.**

**24. Xi Q, He W, Zhang XH, Le HV, Massague J. Genome-wide impact of the BRG1 SWI/SNF chromatin remodeler on the transforming growth factor beta transcriptional program. The Journal of biological chemistry. 2008;283(2):1146-55. Epub 2007/11/16.**

**25. Ross S, Cheung E, Petrakis TG, Howell M, Kraus WL, Hill CS. Smads orchestrate specific histone modifications and chromatin remodeling to activate transcription. The EMBO journal. 2006;25(19):4490-502. Epub 2006/09/23.**

**26. Gebuhr TC, Kovalev GI, Bultman S, Godfrey V, Su L, Magnuson T. The role of Brg1, a catalytic subunit of mammalian chromatin-remodeling complexes, in T cell development. The Journal of experimental medicine. 2003;198(12):1937-49. Epub 2003/12/17.**

**27. Bossen C, Murre CS, Chang AN, Mansson R, Rodewald HR, Murre C. The chromatin remodeler Brg1 activates enhancer repertoires to establish B cell identity and modulate cell growth. Nat Immunol. 2015;16(7):775-84. Epub 2015/05/20.**

**28. Zhou Q, Zhang Y, Wang B, Zhou W, Bi Y, Huai W, et al. KDM2B promotes IL-6 production and inflammatory responses through Brg1-mediated chromatin remodeling. Cellular & molecular immunology. 2020;17(8):834-42. Epub 2019/06/15.**

**29. Vradii D, Wagner S, Doan DN, Nickerson JA, Montecino M, Lian JB, et al. Brg1, the ATPase subunit of the SWI/SNF chromatin remodeling complex, is required for myeloid differentiation to granulocytes. Journal of cellular physiology. 2006;206(1):112-8. Epub 2005/06/21.**

**30. Maher TM, Wells AU, Laurent GJ. Idiopathic pulmonary fibrosis: multiple causes and multiple mechanisms? The European respiratory journal. 2007;30(5):835-9. Epub 2007/11/06.**

**31. Gerstenberger BS, Trzupek JD, Tallant C, Fedorov O, Filippakopoulos P, Brennan PE, et al. Identification of a Chemical Probe for Family VIII Bromodomains through Optimization of a Fragment Hit. Journal of medicinal chemistry. 2016;59(10):4800-11. Epub 2016/04/27.**

**32. Clement S, Juge-Aubry C, Sgroi A, Conzelmann S, Pazienza V, Pittet-Cuenod B, et al. Monocyte chemoattractant protein-1 secreted by adipose tissue induces direct lipid accumulation in hepatocytes. Hepatology. 2008;48(3):799-807. Epub 2008/06/24.**

**33. Fan Z, Sun X, Chen X, Liu H, Miao X, Guo Y, et al. C-C motif chemokine CCL11 is a novel regulator and a potential therapeutic target in non-alcoholic fatty liver disease. JHEP reports : innovation in hepatology. 2023;5(9):100805.**

**34. Puxeddu I, Bader R, Piliponsky AM, Reich R, Levi-Schaffer F, Berkman N. The CC chemokine eotaxin/CCL11 has a selective profibrogenic effect on human lung fibroblasts. J Allergy Clin Immunol. 2006;117(1):103-10. Epub 2006/01/03.**

**35. Kong M, Dong W, Kang A, Kuai Y, Xu T, Fan Z, et al. Regulatory role and translational potential of CCL11 in liver fibrosis. Hepatology. 2023. Epub 2023/01/19.**

**36. Espindola MS, Habiel DM, Narayanan R, Jones I, Coelho AL, Murray LA, et al. Targeting of TAM Receptors Ameliorates Fibrotic Mechanisms in Idiopathic Pulmonary Fibrosis. American journal of respiratory and critical care medicine. 2018;197(11):1443-56. Epub 2018/04/11.**

**37. Yang DC, Gu S, Li JM, Hsu SW, Chen SJ, Chang WH, et al. Targeting the AXL Receptor in Combating Smoking-related Pulmonary Fibrosis. American journal of respiratory cell and molecular biology. 2021;64(6):734-46. Epub 2021/03/18.**

**38. Li W, Xie L, Ma J, Yang M, Wang B, Xu Y, et al. Genetic loss of Gas6/Mer pathway attenuates silica-induced lung inflammation and fibrosis in mice. Toxicology letters. 2019;313:178-87. Epub 2019/07/10.**

**39. Shibata T, Ismailoglu UB, Kittan NA, Moreira AP, Coelho AL, Chupp GL, et al. Role of growth arrest-specific gene 6 in the development of fungal allergic airway disease in mice. American journal of respiratory cell and molecular biology. 2014;51(5):615-25. Epub 2014/05/09.**

**40. Matsushima S, Aoshima Y, Akamatsu T, Enomoto Y, Meguro S, Kosugi I, et al. CD248 and integrin alpha-8 are candidate markers for differentiating lung fibroblast subtypes. BMC pulmonary medicine. 2020;20(1):21. Epub 2020/01/23.**

**41. Hung CF, Wilson CL, Chow YH, Schnapp LM. Role of integrin alpha8 in murine model of lung fibrosis. PloS one. 2018;13(5):e0197937. Epub 2018/05/31.**
